# A randomized trial to monitor the efficacy and effectiveness by QT-NASBA of artemether-lumefantrine versus dihydroartemisinin-piperaquine for treatment and transmission control of uncomplicated *Plasmodium falciparum *malaria in western Kenya

**DOI:** 10.1186/1475-2875-7-237

**Published:** 2008-11-18

**Authors:** Petra F Mens, Patrick Sawa, Sandra M van Amsterdam, Inge Versteeg, Sabah A Omar, Henk DFH Schallig, Piet A Kager

**Affiliations:** 1 Koninklijk Instituut voor de Tropen (KIT)/Royal Tropical Institute, KIT Biomedical Research, Meibergdreef 39, 1105 AZ Amsterdam, the Netherlands; 2 Centre for Infection and Immunity Amsterdam, (CINEMA), Division of Infectious Diseases, Tropical Medicine and AIDS, Academic Medical Centre, Amsterdam, Meibergdreef 9, 1105 AZ Amsterdam, the Netherlands; 3 International Centre for Insect physiology and Ecology (ICIPE), Mbita, Kenya; 4 Kenya Medical Research Institute (KEMRI), Centre for Biotechnology Research and Development, Nairobi, Kenya

## Abstract

**Background:**

Many countries have implemented artemisinin-based combination therapy (ACT) for the first-line treatment of malaria. Although many studies have been performed on efficacy and tolerability of the combination arthemeter-lumefantrine (AL) or dihydroartemisinin-piperaquine (DP), less is known of the effect of these drugs on gametocyte development, which is an important issue in malaria control.

**Methods and results:**

In this two-arm randomized controlled trial, 146 children were treated with either AL or DP. Both groups received directly observed therapy and were followed for 28 days after treatment. Blood samples were analysed with microscopy and NASBA. In comparison with microscopy NASBA detected much more gametocyte positive individuals. Moreover, NASBA showed a significant difference in gametocyte clearance in favour of AL compared to DP. The decline of parasitaemia was slower and persistence or development of gametocytes was significantly higher and longer at day 3, 7 and 14 in the DP group but after 28 days no difference could be observed between both treatment arms.

**Conclusion:**

Although practical considerations could favour the use of one drug over another, the effect on gametocytogenesis should also be taken into account and studied further using molecular tools like NASBA. This also applies when a new drug is introduced.

**Trial registration:**

Current controlled trials ISRCTN36463274

## Background

In response to widespread resistance of *Plasmodium falciparum *parasites to the commonly used drugs chloroquine (CQ) and sulphadoxine-pyrimethamine (SP), many African countries recently adopted artemisinin-based combination therapy (ACT) as first-line treatment for uncomplicated malaria. The combination artemether-lumefantrine (AL) proved to be highly effective and well-tolerated in several studies in Africa [1-5]. Disadvantages of this drug combination are the twice-daily dosing and the fact that it should be administered with a fat-rich meal [6-8] or at least a cup of soya milk [2]. In Uganda, in an area of intense malaria transmission, recurrence of parasitaemia within 28 days occurred in 29% of AL treated patients, in 8.9% adjusted by genotyping, indicating recrudescence [4].

Another ACT, dihydroartemisinin combined with piperaquine (DP), which was originally developed in China, is increasingly used in Southeast Asia [9,10]. Piperaquine is an orally active bisquinoline with a half-life elimination time of 2.5–4 weeks [9]. The drug is structurally related to CQ, but still active against highly CQ-resistant *P. falciparum *strains [1,11]. This relatively inexpensive drug was well tolerated and highly effective in Southeast Asia [1,7,11-13] as was the case in two studies in Africa [4,14]. Consequently, both AL and DP are considered to be amongst the most promising artimisinin-based drugs [4,14]. Artemisinin-based drugs also act on gametocytes and thus on transmission, at least in low transmission areas [15-17]. In high transmission areas of Africa, not much information is yet available on gametocytaemia after ACT treatments and on possible influence on transmission. In a comparative study of AL and DP in Uganda, the appearance of gametocytes in those who did not have gametocytes at the start of treatment was lower from day 15 to day 42 of follow up in those treated with DP than in those treated with AL [4]. A limitation of this study was the fact that gametocytaemia was assessed by microscopic examination only. It has recently been shown that sub-microscopic gametocyte densities may significantly contribute to the transmission of malaria [18].

Adequate assessment of gametocytaemia is important. Quantitative nucleic sequence based amplification technique (QT-NASBA) has been shown to be much more sensitive for the detection of gametocytes than microscopy [3,18-20]. In this study, the post-treatment prevalence of gametocytes in children in Mbita, western Kenya was assessed, after treatment with AL and DP. Microscopy and QT-NASBA for the quantification of gametocytaemia were compared and effectiveness of both drugs regarding clinical symptoms, clearance of parasites and tolerability was assessed.

## Materials and methods

### Study site and population

The study was conducted in Mbita, western Kenya, at the shores of Lake Victoria during the high malaria transmission season of April-July 2007. Mbita, is an area with highly variable transmission that depends on the local environmental circumstances that can support mosquito conditions. The EIR is calculated to be 6 infectious bites per person per month [21]. Children (6 months-12 years of age) visiting the out-patient clinic of the health centre and diagnosed with uncomplicated malaria were included after informed consent from parents or guardians. Inclusion criteria were: uncomplicated *P. falciparum *malaria with initial parasitaemia between 1,000 and < 200,000 parasites/μl blood, axillary temperature ≥ 37.5°C (measured with a digital thermometer) or a history of fever. Children with severe malaria, mixed infection or other underlying illness were excluded from the study. In total 146 children were recruited for the present study. Ethical approval for this study was obtained from appropriate local authorities and The Kenya Medical Research Institute (KEMRI, Nairobi, Kenya) Ethical Steering Committee (SSC protocol No 948). The trial was registered as an International Standard Randomized Controlled Trial at current controlled trials (ISRCTN36463274).

### Study design and treatment

Following diagnosis (at day 0), the patients were randomly allocated to one of the two treatment groups following a computer generated randomization list. One group was assigned DP (Sigma-Tau, Italy) once per day for three days. One tablet of the study drug contained 20 mg of dihydroartemisinin and 160 mg of piperaquine (paediatric formulation). Treatment was according to body weight as follows: children between 4–7 kg received half a tablet per dose, those between 7–13 kg 1 tablet, 13–24 kg 2 tablets per dose and children between 24–35 kg 4 tablets. The other group was assigned to AL (Novartis Pharma, Switzerland). Each tablet contained 20 mg artemether and 120 mg lumefantrine. Patients received treatment according to bodyweight; i.e. children between 5–14 kg received one tablet per dose, those between 15–24 kg two tablets and those between 25–34 kg received three tablets per dose. Doses were given twice daily. All treatments were given with a glass of milk under direct supervision at the clinic or, for the 2^nd ^dose of AL, at home.

### Outcomes: efficacy

Efficacy was assessed using the WHO in vivo test with a follow-up period of 28 days [22]. At enrollment (day 0) a full clinical examination was performed; information was recorded on a case record form. At initial diagnosis (day 0) and during follow-up (day 1, 2, 3, 7, 14, and 28), finger prick blood samples were collected for microscopy, measurement of haemoglobin level and molecular analysis. Haemoglobin was measured with Hemocue 201+ analyser and cuvettes (HemoCue diagnostics B.V. Waarle, The Netherlands). Response to treatment was measured and defined according to WHO guidelines [22].

Patients showing complications or treatment failure were treated with appropriate supportive therapy. Children developing danger signs or severe malaria on day 1 or 2 of the study were withdrawn from the study, referred to the hospital, and given alternative treatment. Adverse events were recorded on the case record forms. An AE was defined as an unfavourable and unintended symptom, sign or disease. A serious adverse event (SAE) was defined as a symptom or sign that is temporally associated with the drugs administered to the patient that is life threatening or results in hospitalization, permanent and significant disability or death. SAE's were immediately reported to the ethical committee of KEMRI and the drug safety department of Sigma-Tau, Italy.

### Outcomes: parasite clearance and gametocyte dynamics

Parasite clearance and gametocyte dynamics were assessed microscopically as well as with quantitative nucleic acid sequence based amplification assay, QT-NASBA.

### Microscopy

Giemsa-stained thick and thin smears were prepared according to WHO guidelines. Two independent experienced microscopists, who were blinded to the treatment and clinical status of the patient, examined the smears for the presence of parasites and identified the observed parasite species. Parasitaemia was determined by counting the number of parasites against 200 leukocytes for the asexual stages (assuming that there are 8,000 leukocytes/μl blood). The presence of gametocytes was examined against 500 leukocytes.

### QT-NASBA

Finger prick blood (50 ul) for NASBA analysis was collected on Whatman 903 filter paper (Whatman international Ltd. Maidston, United Kingdom) and air-dried at room temperature. Nucleic acid extraction was performed as previously described by Boom *et al *[23]. Real-time 18S rRNA QT-NASBA was applied to study asexual parasite clearance below microscopical threshold [20]. In order to quantify the number of parasites in blood, a 10 fold serial dilution of 10^6 ^to 10 in vitro cultured parasites/ml was used as reference and processed and analysed with NASBA. Furthermore, to assess prevalence of gametocytes below the detection limit of microscopy, QT-NASBA targeting Pfs25 mRNA as described by Schneider *et al *[20] was used on blot spots collected during follow-up.

### Genotyping

In order to discriminate between re-infection (RI) and recrudescence (RE), merozoite surface protein 1 and 2 (msp1 and msp2) and glutamate rich protein (GLURP) genotyping was performed as described by Snounou [24] on blood spots obtained at primary (day 0) and secondary infection (time point of re-occurrence). Blood spots were collected on Whatman 903 filter paper (Whatman international Ltd. Maidston, United Kingdom) and air-dried at room temperature for PCR analysis. DNA was isolated as described by Boom *et al *[23]. Molecular analysis was performed at Royal Tropical Institute, Amsterdam and was done blinded from the treatment that was given to the patients.

### Sample size and statistical analysis

The aim of the study was to compare gametocytaemia after AL and DP and to compare assessment of gametocytaemia by microscopical examination versus QT-NASBA All data were entered in excel and analysed with SPSS for windows (version 12.0). Parasite densities were analysed after natural log-transformation. Where appropriate, proportions were compared with the χ^2^-test and means were compared with the one-way ANOVA or Student t-test. A simplified trapezoid area under the curve (AUC) analysis using gametocyte data from days 0, 3, 7, 14 and 28, as a surrogate for the infectiousness of the participants in the different treatment groups, was performed.

## Results

### Patient recruitment

**Figure 1:**
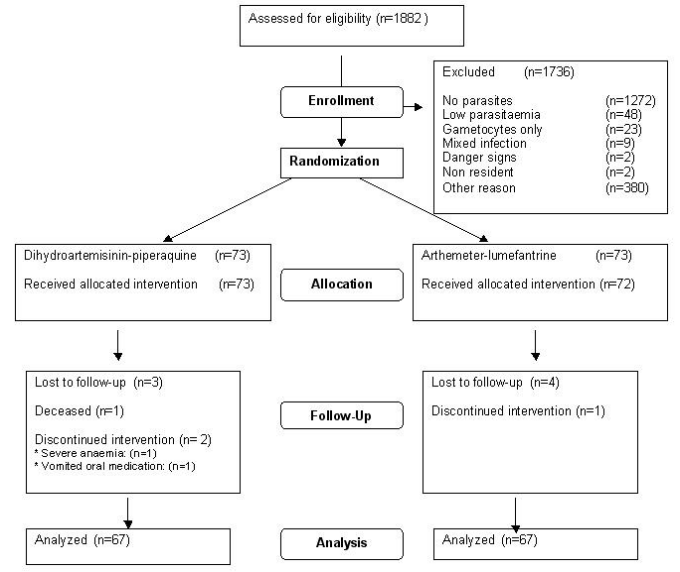
Schematic representation and flowchart of the study.

In total 1882 cases suspected of uncomplicated malaria were screened for eligibility into the study during an 8-week recruitment period in April and May 2007. 1,736 children were excluded because they did not meet the inclusion criteria (Figure 1). 146 patients fulfilling the inclusion criteria entered the study; 73 were randomly allocated to the DP arm and 73 to the AL arm. Both study groups were comparable at baseline for their demographical and clinical characteristics and parasite densities (Table 1). On completion of follow up (day 28) data of 134 patients (92%) were available for analysis.

**Table 1:** Baseline characteristics of patients included in the study at the time of enrollment in the study

**Characteristic**	**DHA-PQP (n = 73)**	**ALN (n = 73)**
Sex ratio male:female	33:40	40:33
Age (in months), median (IQR)	60 (44)	52 (44)
Body weight, mean kg (range)	17.62 (6–37)	17.32 (6–42)
Temperature, mean °C ± SD	38.1 ± 0.99	37.8 ± 0.73
Haemoglobin mmol/L ± SD	6.33 ± 1.29	6.28 ± 1.27
Parasites/μl geometric mean (range) as determined by microscopy	12145 (1000–72640)	13379 (1080–72000)

Twelve patients did not reach the study endpoint. Seven patients were lost during follow up, one was unable to take oral medication, one developed severe anaemia, one did not receive the proper drugs, one withdrew from the study and one patient died.

### Treatment outcome

There were no early treatment failures during the first three days of follow up. Only one patient in the AL arm had a recurrent parasitaemia (43,880 parasites/μl) at day 28 of follow up. Genotyping analysis revealed that this patient had a reinfection with *P. falciparum*. All other 133 patients who completed follow-up had an adequate clinical and parasitological response. After one day of treatment, over 90% of the patients had no microscopically detectable asexual parasites. In the AL group no parasites could be detected with microscopy in any of the patients at day two. One patient was still microscopically positive at day two in the DP group with 40 parasites/μl, but this patient was also microscopically negative at day 3. The parasite reduction ratios at 48 hours reproduction cycle (parasite count on admission/parasite count at 48 hours) was 8.96 * 10^5 ^at 48 hours for the AL treatment and 2.06 * 10^4 ^at 48 hours for the DP treatment.

NASBA was also applied to monitor parasite dynamics below sub-microscopical level. Humidity in some of the filter papers degraded the RNA in the blood spots of some of the samples. This led to several extraction failures. In order to have a clear picture of parasite dynamics only those series with a full range of follow-up samples, i.e. 56 DP and 54 AL treated patients, were analysed. Both treatment arms showed a steep decline in parasitaemia from the day of enrollment (day 0) to day 1; 62% reduction after DP treatment and 89% reduction in the AL arm. At day 2, the level of parasitaemia was reduced to 1.2% in the AL group and 2.75% in the DP group.

### Hb convalescence, fever clearance and adverse events

At baseline Hb levels in both treatment groups were comparable (Table 1). At day 28 all groups had a significant increase of Hb however no significant difference between the treatments on the Hb convalescence was found. Final mean Hb levels were 7.15 mmol/l ± 1.07 for the DP treatment group and 6.79 mmol/l ± 1.24 for the AL group. A possible influence of anaemia on gametocyte carriage at enrollment was not observed in the present study (p > 0.05).

Fever clearance was defined as the time from receiving the assigned treatment to the time a normal body temperature was recorded (≤ 37.5°C), in study cases who presented with fever. Fever clearance was rapid in both study groups. On day 1, 10 cases (13.7%) in the DP group presented with fever and six cases (8.2%) were observed in the AL group. In the DP group fever was observed on day 2 in four cases (5.5%) and three cases (4.1%) on day 3. In the AL group cases with fever also presented on day 2 (12 study subjects, 16.4%) and on day 3 (two individuals, 2.7%). Fever was not observed during follow-up after day 7, with the exception of the child that presented with a *P. falciparum *reinfection on day 28 in the AL group and the child that developed broncho-pneumonia (case presented below). Furthermore the presence of fever at recruitment was no predictor for gametocyte carriage (p > 0.05)

Most adverse events were mild, self limiting and consistent with symptoms of malaria. There was no significant difference between the two study groups (Table 2). One patient died. The child (63 months) had been ill for two weeks prior to presentation at the clinic. *Plasmodium falciparum *infection with parasitaemia of 20,120 parasites/μl was diagnosed. There was a fever (38.6°C), but there were no other complaints and no signs of severe anaemia (Hb: 6.4 mmol/L). On day 3, there were no signs of illness. On day 7, the child presented with fever (38.6°C), cough and complaints of anorexia. There was no history of significant illness or allergies. After examination (microscopy was negative for malaria parasites), broncho-pneumonia was diagnosed and the child was treated with oral phenoxymethylpenicillin for five days and Paracetamol syrup. On day 14, the child did not attend the follow-up visit, the parents reported that the child died a day before in a local health post. The event was assessed as unrelated to the study drug. Autopsy was not performed.

**Table 2:** Summary of adverse events recorded during the study

**Adverse event**	**DHA-PQP**	**ALN**	***P-*value**
Headache	43 (58.9%)	37 (50.7%)	0.318
Abdominal pain	25 (32.4%)	26 (35.6%)	0.862
Weakness	19 (26.0%)	30 (41.1%)	0.035
Anorexia	8 (10.9%)	10 (13.7%)	0.439
Diarrhea	9 (12.3%)	7 (9.6%)	0.785
Cough	16 (21.9%)	17 (23.3%)	0.843
Vomiting	11 (15.1%)	9 (12.3%)	0.806
Pruritis	4 (5.5%)	3 (4.1%)	0.698

### Gametocyte dynamics

The presence of gametocytes in clinical samples was assessed by microscopy and NASBA and is presented in Table 3. At the start of the study, three patients in the DP arm (4.5%) and six patients in the AL arm (9.0%) carried microscopically detectable gametocytes. Microscopical follow-up of the presence of gametocytes during the whole study period revealed that in total 39 samples (distributed over 13 patients) in the DP arm and 18 samples (distributed over nine patients) in the AL arm carried gametocytes (not significantly different). It was observed that the microscopical detection of gametocytes in blood slides during the study was subjected to fluctuations (for example a case positive on day 0, negative on day 1 and subsequently again positive at day 3), which is probably due to the fact that gametocytes circulate at low levels. However, on day 7, three patients in the DP group and 1 in the AL group showed gametocyte positive slides for the first time. On day 28, in none of the cases gametocytes were observed by microscopy. There was no difference between children older than 60 months and younger as regards carriage of gametocytes and density.

**Table 3:** Occurrence of gametocytes as detected by microscopy or NASBA in the different study groups at the start of the study and during subsequent follow-up.

**Gametocyte positive samples**	**Day 0**	**Day 1**	**Day 2**	**Day 3**	**Day 7**	**Day 14**	**Day 28**
**DP group microscopy (n = 67)**							
Total number of positive cases	3	7	7	10	7	5	0
Number of new cases observed		5	2	0	3	0	0

**DP group Nasba (n = 56)**							
Total number of positive cases	22			34^a^	33^a^	17	8
Number of new cases observed				13	8	5	0

**AL group microscopy (n = 67)**							
Total number of positive cases	6	3	3	3	2	1	0
Number of new cases observed		1	1	0	1	0	0

**AL group Nasba (n = 54)**							
Total number of positive cases	21			20	11	12	5
Number of new cases observed				6	4	6	0

^a ^Number of gametocyte carriers detected with NASBA is significantly higher in the DP treated group compared to the AL treated group.

NASBA analysis on 56 DP treated subjects and 54 AL treated subjects detected strikingly more gametocyte carriers at the start of the study compared to microscopy; i.e. 22 study subjects in the DP arm (39.3%) and 21 in the AL (38.9%) were harbouring gametocytes before. The pfs25 NASBA revealed that 34 cases (60.7%) were gametocyte positive in the DP group on day 3, of which 13 cases were newly identified compared to day 0. In contrast, a significantly lower number of patients (20; 37%) were gametocyte positive in the AL treatment group on day 3, of which six new cases. This trend was also observed on day 7: the DP arm had 33 (58.9%) gametocyte positive samples, whereas the AL treated group had a significantly lower number of gametocyte positive samples (11, 20.3%). However on day 14 (AL: 12 positive [22.2%], six new; DP: 17 [30.4%], five new) and day 28 (AL: 5 positive [9.3%], no new cases; DP: 8 [14.3%], no new cases) of follow-up no significant difference in gametocyte carriage was observed between both treatment groups. The AUC _(day 0–28) _of the two treatment groups was calculated to be 20.0 infectious persons/day for the DP treatment arm and 10.5 infectious persons/day for the AL treatment arm. Stratifying the data for age under and above 60 months showed no difference in either of the groups.

## Discussion

Several studies have analysed the efficacy and tolerability of AL and DP and all show very good results [1,4,6,7,12,14,25]. In the present study, the two drugs showed to be similar with respect to effectiveness and tolerability compared to other studies. However, most of these studies have a follow up of 42 days which makes a direct comparison of the results difficult. No adverse events other than those related to malaria itself were observed in the current study, which is in line with other reports. In the present study, all children experienced haemoglobin convalescence without difference between the two treatment arms, in contrast to the study of Kamya *et al*, who found a greater increase in the DP treated patients [4]. The difference in follow-up time and the numbers of patients included in both studies may be responsible for this difference. Further studies with comparable study length should be done to give an answer to these discrepancies.

The effects on gametocytaemia and possibly malaria transmission deserve further study. Whereas asexual parasites were cleared in three days after the initiation of the two treatment schedules, gametocytaemia appeared different when assessed by microscopy as well as with NASBA. Gametocytes were present in low numbers throughout follow-up in both study groups. Artemisinin derivatives have in general a negative effect on gametocyte development and survival and thus influence malaria transmission, at least in low transmission areas [15-17]. In this study, the actual infectiousness of the remaining gametocyte populations in both treatment arms was not assessed; the presence of gametocytes does not necessarily mean that they actually contribute to transmission. Several studies have shown that gametocytes persist in a large population of previously infected and treated children [5,19,26,27]. A large proportion of these carriers has a parasite load below microscopical detection limit, a load that can be detected with molecular assays like NASBA. Patients with submicroscopic parasite densities may still be infectious to mosquitoes and may contribute to transmission [14,19], as confirmed with membrane feeding experiments [18,28]. Studies that include reduction of transmission as a component of efficacy of drugs, thus need to incorporate highly sensitive molecular assays to reliably assess gametocyte densities.

The present study showed a limited effect of DP on gametocyte development in comparison with AL when a sensitive tool like NASBA is used for gametocyte detection, which could limit the usefulness of DP to areas with low transmission but this finding should be further investigated in larger studies in different study sites with different transmission intensities. It is not clear if plasma concentrations of dihydroartemisinin in the blood could play a role. Dihyrodartemisinin is the major and the active metabolite of artemether. So far, no studies have been performed that compare the plasma levels of dihydroartemisinin when given as such or after administration of artemether and subsequent metabolisation. This should be further investigated together with effect on gametocytogenesis, which should incorporate a sensitive detection tool for gametocytes such as NASBA.

The effect that drugs can have on gametocyte clearance as measured with NASBA could have some implications for the introduction drugs and especially the introduction of new drugs. This study showed that with sensitive detection tools a difference in parasite clearance can be observed but these results should be confirmed in larger studies and in other study areas with different malaria transmission intensities. Transmission intensity varies significantly in the different African countries and within a country high and low transmission areas can often be identified. Malaria endemic countries generally have a national malaria drug policy for the whole country. Although this is logical from a practical and logistical point of view, it may not be the best approach for effective malaria control. It could, therefore, be more effective if a country develops specific drug policies to suit regional instead of national requirements.

## Competing interests

The authors declare not to have a conflict of interest. The organizations that provided financial support had no influence in the design, the actual field and laboratory work, analysis and writing.

## Authors' contributions

PM was involved in the conception of the study, carried out the molecular analysis of the samples, carried out the statistical analysis of the results and drafted the manuscript. PS coordinated the study in the field and was responsible for the clinical examination and treatment of the participants. SvA collected clinical data and blood samples in the field and did the clinical data analysis. SO coordinated the study in the field an arranged the logistics in the field. IV carried out the NASBA assays and the subsequent analysis.

HS was involved in the design of the study and contributed to the drafting of the manuscript. PK as involved in the design of the study, critically read and improved the manuscript. All authors read and approved the final manuscript
